# Age-stratified clinical characteristics and long-term neurological outcomes of pediatric herpes simplex virus encephalitis: a longitudinal cohort study of 56 children

**DOI:** 10.3389/fneur.2026.1882046

**Published:** 2026-07-23

**Authors:** Ying Wang, Yanli Ma, Yuan Wang, Zhixiao Yang, Jun Zhang, Chunge Li, Shijie Dong, Weihua Zhang, Yufeng Liu

**Affiliations:** 1 Department of Pediatrics, The First Affiliated Hospital of Zhengzhou University, Zhengzhou, China; 2 Department of Neurology, Children’s Hospital Affiliated to Zhengzhou University, Zhengzhou, China; 3 Department of Medical Imaging, Children’s Hospital Affiliated to Zhengzhou University, Zhengzhou, China; 4 Department of Neurology, National Centre for Children’s Health, Beijing Children’s Hospital, Capital Medical University, Beijing, China

**Keywords:** age-stratified, anti-NMDAR encephalitis, children, facial twitching, herpes simplex virus encephalitis, prognosis

## Abstract

**Background and objective:**

Age-specific clinical characteristics and prognostic predictors of pediatric herpes simplex virus encephalitis (HSE) remain incompletely characterized. This study aimed to characterize age-dependent manifestations and identify factors independently associated with poor long-term outcomes.

**Methods:**

A longitudinal cohort study included 56 pediatric patients with confirmed HSE who were admitted to the Children’s Hospital Affiliated to Zhengzhou University from January 2018 to June 2023. Patients were stratified into three age groups: infant/toddler group (0–3 years), preschool group (3–6 years), and school-age and older group (6–18 years). Clinical, laboratory, and cranial magnetic resonance imaging (MRI) data were collected. Neurological outcomes were assessed using the pediatric modified Rankin Scale (mRS). Multivariate logistic regression analysis was performed to identify independent associated factors of poor prognosis.

**Results:**

The median age of onset was 2.09 years, and 60.7% of patients were aged under 3 years. Unilateral facial and/or perioral twitching occurred in 33.9% of patients, all of whom under 5 years of age, with the highest incidence in the preschool group (70.0%, *p* = 0.001). This sign was associated with higher rates of frontal/insular lobe involvement and severe long-term language impairment (89.5% vs. 51.4%, *p* = 0.005). The median follow-up duration was 62.5 months. Overall, 73.2% of patients had a poor outcome. The incidence of post-encephalitic epilepsy (PE) was 53.6%, of which 63.3% were drug-refractory. Anti-N-methyl-D-aspartate receptor (NMDAR) encephalitis developed in 17.9% of patients, and its incidence increased significantly with age (*p* = 0.002). Multivariate logistic regression analysis identified a lower acute Glasgow Coma Scale (GCS) score (OR = 0.577, 95% CI: 0.376–0.885, *p* = 0.012) and prolonged total seizure duration (OR = 1.635, 95% CI: 1.126–2.374, *p* = 0.010) as factors independently associated with poor prognosis.

**Conclusion:**

Pediatric HSE exhibits notable age-associated heterogeneity. A low acute GCS score and prolonged seizure duration were identified as independent associated factors for poor prognosis in this exploratory analysis, whereas early facial/perioral twitching was associated with subsequent language sequelae. The risk of anti-NMDAR encephalitis increases with age, necessitating long-term surveillance. These age-stratified findings are descriptive and hypothesis-generating and require confirmation in larger prospective cohorts.

## 1 Introduction

Herpes simplex virus encephalitis (HSE) is the most common and severe form of sporadic viral encephalitis, with an annual incidence of approximately 1/250,000–1/500,000 (1, 2). About one-third of cases occur in children (3–5). Although acyclovir has significantly reduced acute mortality of HSE (6–8), a considerable proportion of survivors suffer from long-term neurological sequelae (9, 10), imposing a heavy burden on families and society. A French multicenter study reported that 76% of pediatric HSE survivors had neurological deficits, including epilepsy (57%), intellectual disability (51%), and language impairment (47%) (11).

Current knowledge regarding the clinical features of HSE is derived mainly from adult studies or mixed-age cohorts. However, the pediatric central nervous system is dynamically developing, with large differences in immune maturity, myelination, and neuroplasticity across age groups. Systematic comparisons of age-specific clinical phenotypes of pediatric HSE are lacking in the literature. Specifically, the following questions remain unanswered: (1) Do clinical manifestations differ significantly between infants, preschool children, and school-aged children? (2) Which neuroimaging features independently predict long-term neurological outcomes? (3) Are there distinctive clinical signs that could improve early diagnosis in young children?

To address these gaps, we analyzed 56 pediatric HSE cases. Through age stratification, we systematically describe the clinical, imaging, and long-term outcome characteristics of different age groups, aiming to improve early recognition and provide evidence for individualized treatment and long-term management.

## 2 Materials and methods

### 2.1 Study design and patients

In this longitudinal cohort study, pediatric patients with confirmed HSE who were hospitalized at the Children’s Hospital Affiliated to Zhengzhou University between January 2018 and June 2023 were enrolled. The date of HSE diagnosis served as the starting point of the study cohort. All enrolled patients or their legal guardians provided written informed consent for the use of their clinical data upon admission. This study was approved by the Ethics Committee of the Children’s Hospital Affiliated to Zhengzhou University.

Inclusion criteria were as follows: (1) age 0–18 years; (2) altered mental status lasting >24 h; and at least three of the following criteria (9, 12): fever (temperature ≥38 °C) within 72 h of onset, new-onset generalized or focal seizures, new focal neurological deficit, cerebrospinal fluid (CSF) white blood cell count ≥5 × 10^6^/L, neuroimaging evidence of brain parenchymal lesions, or electroencephalography (EEG) abnormalities consistent with encephalitis; (3) positive CSF herpes simplex virus type 1 (HSV-1) nucleic acid detected by polymerase chain reaction (PCR) or metagenomic next-generation sequencing (mNGS).

Exclusion criteria were as follows: (1) incomplete clinical data; (2) other pre-existing central nervous system diseases before HSE onset; (3) no brain magnetic resonance imaging (MRI) was completed within 1 month of onset; (4) less than 1 year of follow-up.

### 2.2 Data collection

#### 2.2.1 Clinical data collection

Demographic data, clinical symptoms, and laboratory data, including CSF examination, EEG, and cranial MRI, were recorded. The level of consciousness was assessed using the Glasgow Coma Scale (GCS). All patients were divided into three age groups: infant/toddler group (0–3 years), preschool group (3–6 years), and school-age and older group (6–18 years). These clinical parameters were subsequently compared among the groups.

Neurological prognosis was assessed at the last follow-up using the pediatric modified Rankin Scale (mRS) (13). A good outcome was defined as mRS score ≤2. After discharge, all patients were scheduled for regular follow-up assessments at 3 and 12 months post-discharge, and annually thereafter. Follow-up evaluations were conducted either through in-person visits at our pediatric neurology outpatient clinic or, for patients who were unable to attend in person due to geographic or other constraints, through structured telephone interviews conducted by trained pediatric neurologists. Two experienced pediatric neurologists, blinded to the acute-phase clinical and imaging data, independently performed the assessments; inter-rater agreement was high. Death was scored as mRS = 6 and included in the poor-outcome group (mRS > 2). For patients with variable follow-up durations, the last available mRS score was used for the primary analysis. During follow-up, the following neurological outcomes were recorded: anti-N-methyl-D-aspartate receptor (NMDAR) encephalitis, HSE recurrence, and the presence of neurological sequelae, including intellectual disability, motor dysfunction, and epileptic seizures.

#### 2.2.2 Pathogen detection and management

A standardized etiological workup was performed for all patients upon admission. The routine encephalitis pathogen panel included HSV-1/2, Varicella-zoster virus (VZV), Epstein–Barr virus (EBV), Cytomegalovirus (CMV), Enteroviruses, *Mycoplasma pneumoniae*, and bacterial cultures of CSF. In cases with negative HSV-PCR but high clinical suspicion, mNGS of CSF was performed. All enrolled patients had confirmed HSV-1 infection, and no concurrent neuroinvasive co-infections were identified.

All patients received intravenous acyclovir (10–15 mg/kg every 8 h) for 14–21 days, adjusted according to renal function. In addition to antiviral therapy, management included: (1) Intracranial pressure (ICP) control. Osmotic therapy with 20% mannitol (0.5–1.0 g/kg per dose) was administered to patients with clinical or radiological signs of cerebral edema. (2) Antibiotic therapy. Empirical broad-spectrum antibiotics were initiated when bacterial co-infection was suspected and discontinued upon negative culture results. (3) Hemodynamic and respiratory support. Vasopressors were administered for refractory shock, and mechanical ventilation was provided for patients with respiratory failure or severely impaired consciousness.

Intensive care unit (ICU) admission criteria were pre-defined as follows: (1) refractory status epilepticus, (2) need for invasive mechanical ventilation, or (3) hemodynamic instability requiring continuous vasopressor infusion.

Anti-NMDAR antibody testing was performed during the convalescent phase when patients developed new or worsening neurological symptoms suggestive of autoimmune encephalitis (including abnormal mental/behavioral status, movement disorders, new-onset seizures, or cognitive decline). Testing was conducted using a cell-based assay (EUROIMMUN, Germany) on serum and/or cerebrospinal fluid samples. This testing protocol was symptom-triggered rather than systematic, which we acknowledge may introduce ascertainment bias. The cumulative incidence of anti-NMDAR encephalitis was estimated using Kaplan–Meier analysis, with patients censored at their last follow-up visit.

#### 2.2.3 Imaging data collection

Cranial MRI was performed on all patients using Siemens 3-T or 1.5-T scanners. The imaging sequences included T1-weighted imaging (T1WI), T2-weighted imaging (T2WI), fluid-attenuated inversion recovery (FLAIR), and diffusion-weighted imaging (DWI) sequences. A scoring system was applied to the left and right cerebral hemispheres, covering five cortical lobes (frontal, parietal, temporal, occipital, and insular) as well as the thalamus. Normal signal intensity was assigned a score of 0, and abnormal signal intensity a score of 1, yielding a theoretical total score range of 0 to 12 per patient. This scoring system was based on the anatomical atlas (Supplementary Figure 1). Two experienced radiologists who were blinded to the clinical outcomes independently performed the scoring. Any disagreement was resolved through discussion until consensus was reached. The presence of hemorrhagic changes was also recorded.

### 2.3 Definitions

Postencephalitic epilepsy (PE) was defined as the continued use of antiseizure medications (ASMs) for ≥12 months after recovery from the acute phase of HSE, including cases in which ASMs were newly initiated and continued beyond the 12-month time point (10). Status epilepticus (SE) was defined as continuous seizure activity lasting more than 5 min, or recurrent seizures within 5 min without a return to baseline consciousness between seizures (3). Convulsive seizure duration was defined as the total calendar days from the first to the last convulsive seizure during the acute phase, with the cessation of seizure activity defined as a seizure-free interval of at least 24 h between episodes.

### 2.4 Statistical analysis

Continuous variables with a normal distribution were expressed as the mean ± standard deviation (SD) and compared using the t-test. Those without a normal distribution were presented as the median with interquartile range (IQR) and compared using the Mann–Whitney U test. Categorical variables were expressed as frequencies and percentages and compared using the chi-square test or Fisher’s exact test, as appropriate. Variables with *p* < 0.10 in univariate analysis were entered as candidates into a backward stepwise multivariate logistic regression model (Likelihood Ratio method) to identify independent predictors of poor long-term prognosis. The entry criterion was set at *p* < 0.05 and the removal criterion at *p* > 0.10. The final model was selected based on the likelihood ratio test, with results presented as exploratory given the single-center retrospective design. Model calibration was assessed using the Hosmer-Lemeshow goodness-of-fit test, and discrimination was evaluated using Nagelkerke R^2^. The events-per-variable (EPV) ratio was calculated to assess model stability. Age-stratified analysis served as the primary analytical strategy. All statistical analyses were performed using IBM SPSS 23.0, ORIGIN 2022, and GraphPad Prism 8. A *p*-value < 0.05 was considered statistically significant.

For descriptive purposes, we explored optimal cut-offs for the number of DWI-involved lobes and total seizure duration using receiver operating characteristic (ROC) curve analysis with Youden index maximization (Youden index = sensitivity + specificity − 1). The choice of the ≥7 lobes threshold was informed by a previous study demonstrating that extensive multilobar DWI involvement is associated with poor prognosis in severe HSE (12). These cut-offs were derived from our own cohort data and are presented as exploratory; they require validation in independent cohorts. In the regression analyses, we prioritized the continuous forms of these variables (number of lobes and seizure duration in days) to avoid information loss from dichotomization. The dichotomized versions were used only for descriptive subgroup comparisons.

All subgroup analyses and lobe by lobe comparisons were exploratory in nature. To address the risk of type I error arising from multiple comparisons, we applied Bonferroni correction to the 9 MRI related variables in the subgroup analyses (frontal, temporal, parietal, occipital, insular, thalamus, ≥7 lobes, bilateral lesions, and hemorrhage), with a corrected significance threshold of *α* = 0.05/9 = 0.0056. Bonferroni-adjusted *p* values are presented in Supplementary Table 1 for transparency. All such findings should be interpreted as descriptive and hypothesis generating, and require validation in independent cohorts. The primary prespecified comparisons (twitching vs. non-twitching groups, and the parsimonious multivariable model) were not adjusted, as they were limited in number and hypothesis driven.

## 3 Results

### 3.1 Clinical manifestations

A total of 56 pediatric patients who met the inclusion criteria were enrolled in this study, including 27 males (48.2%) and 29 females (51.8%). There was no significant difference in sex distribution among the age groups. The median age at onset was 2.09 years (IQR: 0.95–4.96 years), with children under 3 years of age accounting for 60.7% (34/56). The most common presenting symptoms included fever (55/56, 98.2%), seizures (50/56, 89.3%), and impaired consciousness (31/56, 55.4%). Focal neurological deficits were also frequently observed, including hemiparesis (53.6%), speech impairment (67.9%), and dysphagia (66.1%). Headache (7.1%), vomiting (17.9%), and abnormal mental status and behavior (10.7%) were relatively rare. The incidence of SE was 35.7% (20/56). The median GCS score on admission was 7 (IQR: 5.25–9).

**Figure 1:**
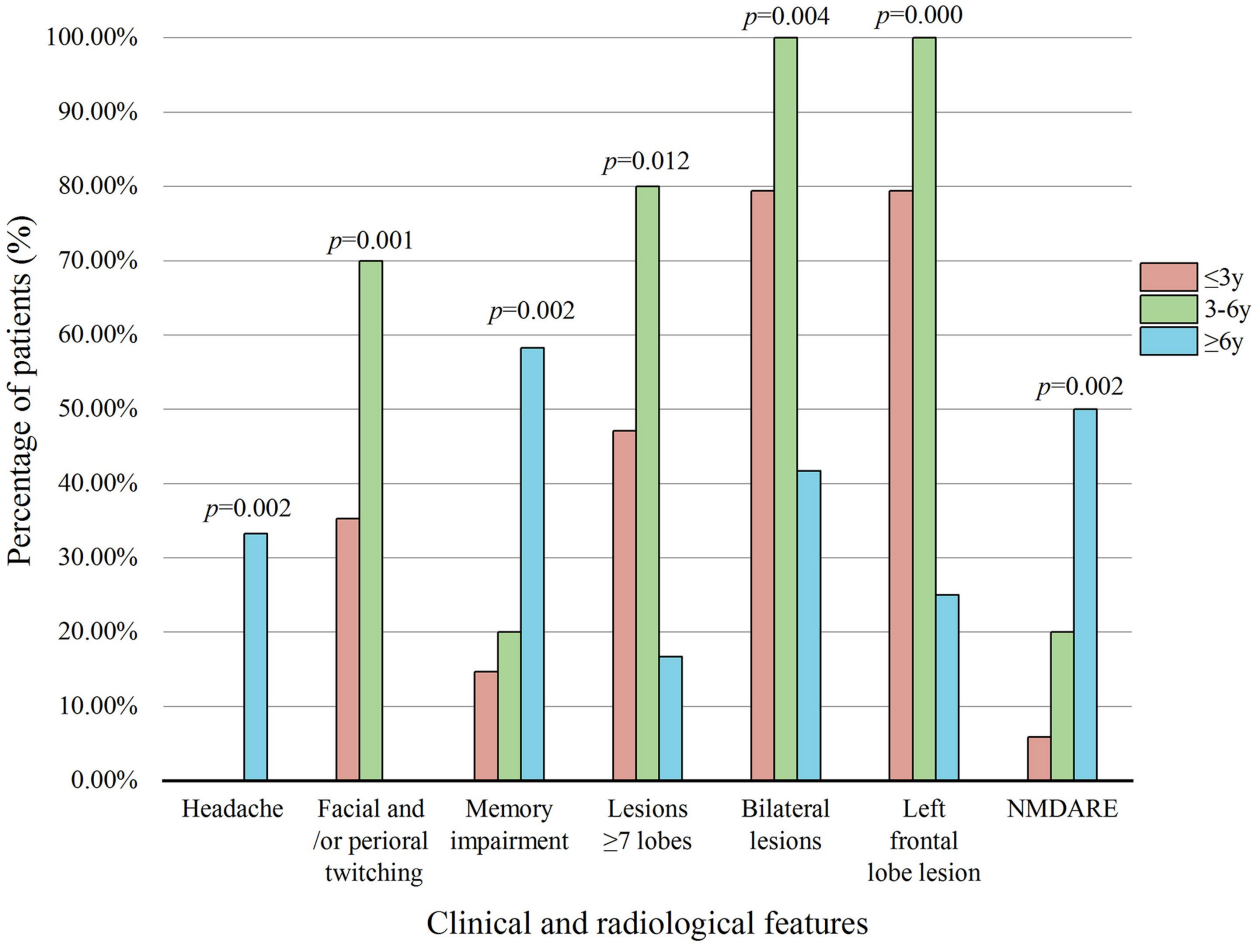
Age-stratified clinical characteristics, imaging features, and outcomes of pediatric HSE.

The patients were divided into three age groups: infant/toddler group (*n* = 34), preschool group (*n* = 10), and school-age and older group (*n* = 12). Given the small sample sizes in the preschool (*n* = 10) and school-age (*n* = 12) subgroups, all age-stratified comparisons are presented as descriptive and hypothesis-generating. Percentages are reported with denominators to facilitate accurate interpretation, and *p*-values are provided for exploratory reference only. Clinical characteristics showed age-associated trends in this exploratory subgroup analysis (Figure 1). Unilateral facial and/or perioral twitching was most common in the preschool group (70.0%, 7/10), whereas this manifestation was not observed in the school-age and older group (*p* = 0.001). The incidence of headache and memory impairment increased with age (*p* = 0.002). The GCS score was lowest in the preschool group (median 6) and highest in the school-age and older group (median 8.5), with a statistically significant difference (*p* = 0.026).

All patients (100%) exhibited abnormalities on cranial MRI. On acute-phase DWI, signal abnormalities were most commonly observed in the temporal lobe (89.3%), parietal lobe (87.5%), frontal lobe (82.1%), and thalamus (85.7%). In addition to cortical/subcortical involvement, white matter hyperintensities were present in 29 patients (51.8%), and meningeal hyperintensity was observed in 6 patients (10.7%). Midline shift was identified in 6 patients (10.7%), all of whom had extensive unilateral lesions with significant mass effect. Extensive DWI signal abnormalities (exploratory cut-off of ≥7 lobes derived via ROC/Youden analysis) were identified in 46.4% of patients. The preschool group had the highest median number of lobes with DWI signal abnormalities, with 80% of patients showing lesions in more than 7 lobes (8/10), followed by the infant/toddler group (47.1%) and the school-age and older group (16.7%, *p* = 0.012). Bilateral damage on brain MRI was nearly universal in the preschool group (100%, 10/10), whereas it was observed in only 41.7% of the school-age and older group (*p* = 0.016). After Bonferroni correction, none of the lobe specific MRI variables reached statistical significance at the corrected level, although bilateral lesions showed a borderline trend (adjusted *p* = 0.036). All lobe-specific findings should therefore be interpreted with caution as exploratory.

**Figure 2:**
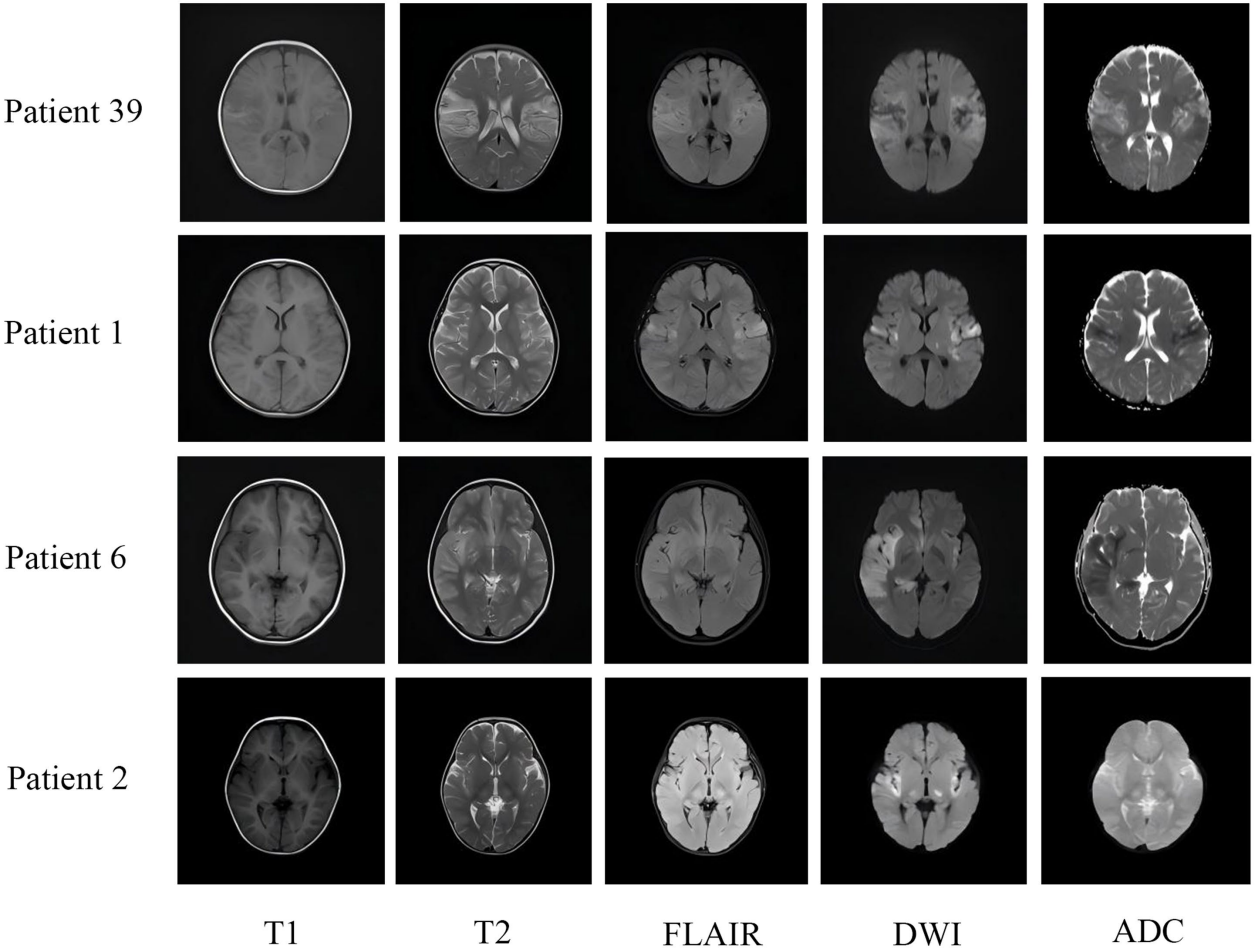
Representative cranial MRI findings in pediatric HSE across different age groups. Patient 39: An 8-month-old female with symmetric temporal lobe lesions and hemorrhage. Patient 1: A 3-year-11-month-old female with bilateral frontal, temporal, parietal, and insular lesions and left thalamic involvement, presenting with perioral/facial twitching during the acute phase. Patient 6: A 10-year-old female with anti-NMDAR encephalitis secondary to HSE, showing right fronto-temporo-parietal and bilateral insular involvement. Patient 2: A 1-year-3-month-old female with Lennox-Gastaut syndrome (LGS) as a sequela of HSE, showing bilateral extensive cortical and thalamic lesions. These images illustrate the age-dependent heterogeneity in lesion distribution and clinical evolution.

The illustrative cases in Figure 2 were selected to represent the main age-dependent imaging patterns observed in our cohort: (1) an 8-month-old infant with symmetric temporal lobe lesions and hemorrhage (Patient 39), representing the infantile pattern of bilateral limbic involvement; (2) a 3-year-11-month-old child with extensive fronto-temporo-parietal lesions and perioral twitching (Patient 1), illustrating the opercular predilection in preschool children; (3) a 10-year-old girl with secondary anti-NMDAR encephalitis (Patient 6), demonstrating the age-related shift toward autoimmune complications; and (4) a 1-year-3-month-old with Lennox–Gastaut syndrome (Patient 2), representing severe post-encephalitic sequelae. These cases collectively highlight the age-dependent heterogeneity in lesion distribution and long-term outcomes, reinforcing the main message of our study.

At the time of hospital discharge, 12 patients (21.4%) had a favorable outcome (mRS ≤ 2), while 44 patients (78.6%) had an unfavorable outcome (mRS > 2). Among those with unfavorable outcomes at discharge, the predominant deficits included motor dysfunction (*n* = 28, 50.0%), dysphagia (*n* = 33, 58.9%), and language impairment (*n* = 31, 55.4%). In-hospital mortality was 0% (no deaths during the acute hospitalization). Notably, the majority of patients with poor short-term outcomes at discharge also had poor long-term prognosis (93.2%, 41/44), confirming the strong correlation between short-term and long-term outcomes.

The median follow-up duration was 62.5 months (IQR: 38.3–78.0 months). At the last follow-up, 41 patients (73.2%) had a poor prognosis (mRS > 2), including 2 patients (3.6%) who died during follow-up (mRS = 6). A sensitivity analysis restricted to patients with ≥24 months of follow-up (n = 38) yielded a similar poor-outcome rate (73.7%, 28/38). PE developed in 30 patients (53.6%), of whom 14 (25%) presented with epileptic spasms at a median age of 2.18 years (IQR: 0.87–3.46 years); two of these patients progressed to Lennox–Gastaut syndrome (LGS). Ten patients (17.9%) developed anti-NMDAR encephalitis during follow-up, diagnosed at a median of 31.5 days (IQR: 26–36.25 days) after HSE onset. The Kaplan–Meier estimated cumulative incidence at 5 years was 18.2%, consistent with the crude incidence. A sensitivity analysis restricted to patients with ≥24 months of follow-up (*n* = 38) yielded a similar incidence of 18.4% (7/38), suggesting that differential follow-up duration did not substantially bias the estimate. Other neurological sequelae included speech impairment in 36 patients (64.3%), motor dysfunction in 23 patients (41.1%), and cognitive impairment in 43 patients (76.8%).

The clinical manifestations, imaging features, and outcomes by age are detailed in Supplementary Table 1.

### 3.2 Facial and/or perioral twitching

During the acute phase, 19 patients (33.9%) presented with unilateral facial and/or perioral twitching, all of whom were under 5 years of age. Compared with the non-twitching group, the twitching group showed a higher proportion of lesions in the left frontal lobe (100% vs. 56.8%, exploratory analysis, *p* = 0.001), right frontal lobe (89.5% vs. 62.2%, *p* = 0.032), left insular lobe (84.2% vs. 56.8%, *p* = 0.040), and bilateral lesions (100% vs. 62.2%, *p* = 0.002). The proportion of patients with lesions in more than 7 lobes was also higher in the twitching group (68.4% vs. 35.1%, exploratory analysis, *p* = 0.018). At follow-up, the twitching group showed a higher proportion of severe language impairment (89.5% vs. 51.4%, exploratory analysis, *p* = 0.005). However, there were no statistically significant differences between the two groups in terms of poor prognosis (mRS > 2), dysphagia, intellectual disability, and PE (Table 1).

**Table 1:** Comparison of imaging features and prognosis between children with and without facial/perioral twitching.

Variables	Facial/perioral twitching group (*n* = 19)	Non-twitching group (*n* = 37)	*p*
Female, *n* (%)	10 (52.6)	19 (51.4)	0.928
Age, years, median (IQR) [range]	2.68 (0.93, 3.58) [0.59–4.97]	1.97 (1.04, 7.57) [0.05–13.24]	0.574
DWI hyperintensity, *n* (%)			
Left frontal lobe	19 (100)	21 (56.8)	0.001
Right frontal lobe	17 (89.5)	23 (62.2)	0.032
Left Insula	16 (84.2)	21 (56.8)	0.040
Right Insula	14 (73.7)	21 (56.8)	0.215
Lesions ≥7 lobes	13 (68.4)	13 (35.1)	0.018
Bilateral lesions	19 (100)	23 (62.2)	0.002
Outcome, *n* (%)			
mRS>2	16 (84.2)	25 (67.6)	0.183
Language impairment	17 (89.5)	19 (51.4)	0.005
Dysphagia	3 (15.8)	1 (2.7)	0.108
Intellectual disability	14 (73.7)	29 (78.4)	0.745
Post-encephalitic epilepsy	9 (47.4)	21 (56.8)	0.505

Data are represented as numbers (percentage) or median (interquartile range).

DWI, diffusion-weighted imaging; mRS, modified Rankin Scale; IQR, interquartile range.

Left and right sided lobar data are presented separately in this table to evaluate potential lateralization associated with facial/perioral twitching, as the opercular cortex origin may have hemispheric predominance. All patients in the twitching group (*n* = 19, 100%) were under 5 years of age, as detailed in Supplementary Table 1. All lobe‑specific comparisons in this table are exploratory and have not been adjusted for multiple comparisons. Bonferroni‑adjusted analyses are presented in Supplementary Table 1 for the overall cohort.

### 3.3 Laboratory characteristics

All patients underwent CSF examination during the acute phase. Elevated CSF white blood cell count was observed in 47 patients (83.9%), with a median value of 20 × 10^6^/L (range: 0–449 × 10^6^/L). Elevated CSF protein was found in 16 patients (28.6%), with a maximum value of 1,394 mg/L. Neither CSF white blood cell count nor protein level was significantly correlated with prognosis (both *p*>0.05).

**Figure 3:**
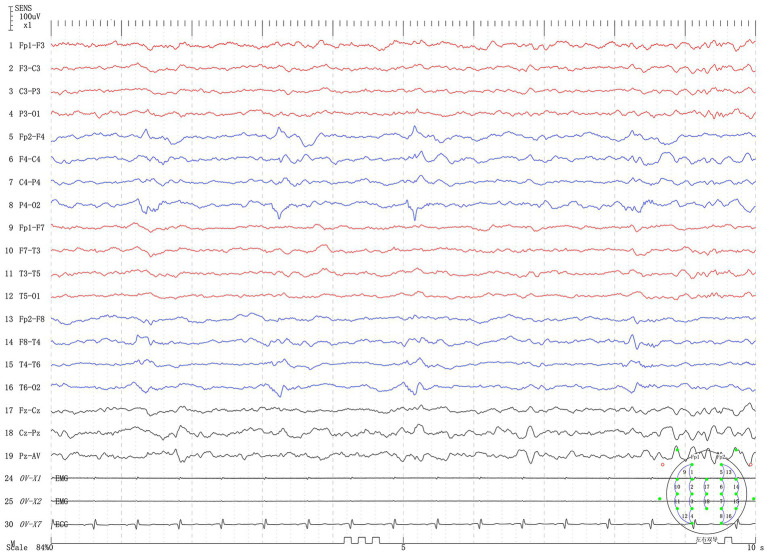
Right-sided periodic lateralized epileptiform discharges in a child with HSE.

All patients had abnormal EEG findings during the acute phase. Diffuse slow waves were present in 47 patients (83.9%), focal slow waves in 5 patients (8.9%), and diffuse low voltage with disappearance of sleep spindles in four patients. Focal epileptiform discharges were observed in 16 patients (28.6%), and periodic lateralized epileptiform discharges (PLEDs) were seen in 5 patients (8.9%) (Figure 3). Seizures were captured on EEG in 12 patients, all of whom had electroclinical seizures of focal onset: temporal lobe onset in 7 cases, frontal lobe onset in 3 cases, and occipital lobe onset in 2 cases.

### 3.4 Factors associated with poor outcome

To identify optimal cut-offs for descriptive subgroup comparisons, we performed ROC curve analysis with Youden index maximization. For the number of DWI-involved lobes, the analysis yielded an area under the curve (AUC) of 0.737 (95% CI: 0.602–0.871), with a Youden index of 0.392 for the ≥7 lobes cut-off. For total seizure duration, the ROC analysis yielded an AUC of 0.819 (95% CI: 0.684–0.953, *p* < 0.001), with a maximum Youden index of 0.580 at a cut-off of >4.5 days, which was rounded to >5 days for clinical interpretability. These cut-offs were used only for descriptive subgroup comparisons and are exploratory; they require external validation in independent cohorts.

Univariate analysis showed that the factors associated with poor prognosis included younger age (*p* = 0.024), ICU admission (*p* = 0.035), dysphagia (*p* = 0.019), low admission GCS score (*p* = 0.015), prolonged duration of convulsive seizures in the acute phase (*p* = 0.001), number of involved lobes on DWI ≥ 7 (*p* = 0.001), symmetric lesion distribution (*p* = 0.016), and involvement of the left temporal lobe (*p* = 0.031), and left parietal lobe (*p* = 0.017) (Table 2).

**Table 2:** Comparison of clinical data of patients with good and poor outcomes.

Variables	Good outcome *n* = 15	Poor outcome *n* = 41	*p*
Age, years, median (IQR)	2.74 (0.92, 7.40)	2.21 (1.03, 3.62)	0.024
ICU admission, *n* (%)	7 (46.7)	34 (82.9)	0.035
Dysphagia, *n* (%)	4 (26.7)	29 (70.7)	0.019
SE, *n* (%)	5 (33.3)	15 (36.6)	0.850
Duration of convulsive seizures, days; mean ± SD	2.87 ± 2.23	5.9 ± 2.85	0.001
GCS score, mean ± SD	8.4 ± 1.88	6.48 ± 1.71	0.015
Time to acyclovir initiation, days; mean ± SD	5.07 ± 2.60	4.9 ± 4.57	0.390
DWI hyperintensity, *n* (%)			
Lesions ≥7 lobes	2 (13.3)	26 (63.4)	0.001
Bilateral lesions	8 (53.3)	36 (87.8)	0.016
Left temporal lobe involvement	8 (53.3)	35 (85.4)	0.031
Left parietal lobe involvement	6 (40)	32 (78)	0.017

Data are represented as numbers (percentage), mean ± standard deviation, or median (interquartile range).

IQR, interquartile range; SD, standard deviation; ICU, intensive care unit; SE, status epilepticus; GCS, glasgow coma scale; DWI, diffusion-weighted imaging.

**Figure 4:**
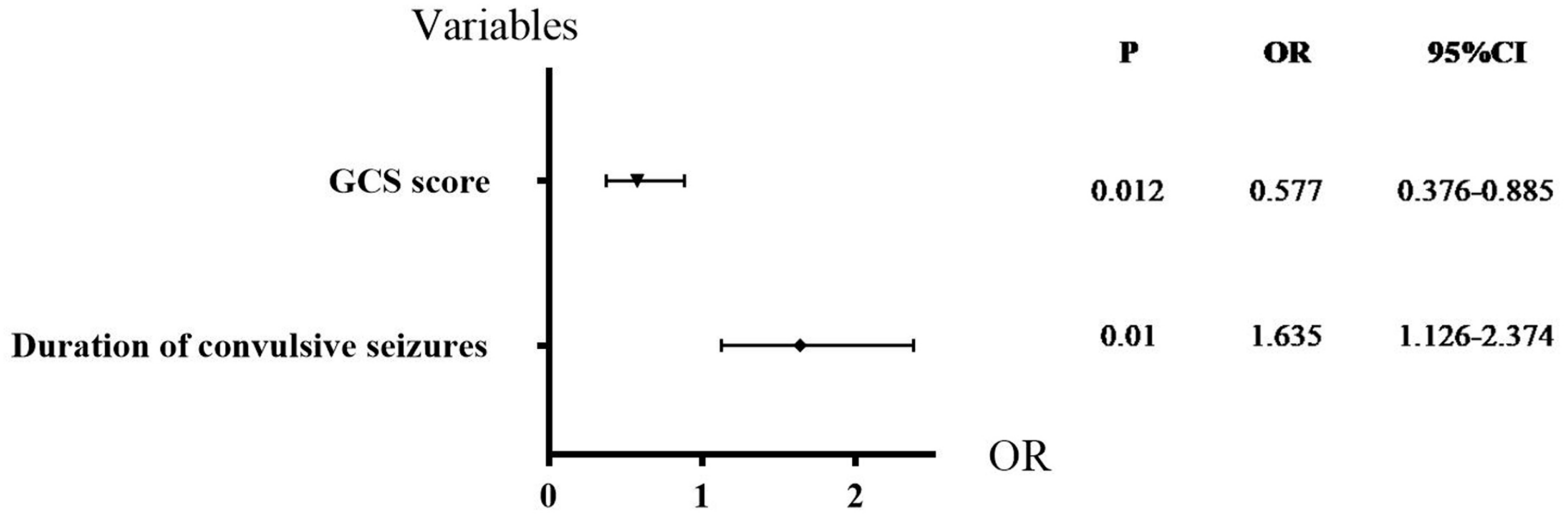
Forest plot of the final parsimonious multivariable logistic regression model for predictors of poor long-term outcome.

Backward stepwise logistic regression (Likelihood Ratio method) was performed with all 9 variables showing *p* < 0.10 in univariate analysis as candidates. After stepwise elimination, the final parsimonious model retained two variables independently associated with poor prognosis: acute GCS score (OR = 0.577, 95% CI: 0.376–0.885, *p* = 0.012), and total duration of convulsive seizures (OR = 1.635, 95% CI: 1.126–2.374, *p* = 0.010) (Figure 4). The final model demonstrated good discrimination, with a Nagelkerke R^2^ of 0.557, and satisfactory calibration (Hosmer-Lemeshow test: χ^2^ = 10.472, df = 7, *p* = 0.163). With 41 poor-outcome events and 2 predictors retained, the EPV ratio was approximately 20.5, meeting the recommended threshold of ≥10. These results should be interpreted as exploratory, given the sample size constraints and the single-center retrospective design.

## 4 Discussion

This study analyzed 56 etiologically confirmed pediatric HSE cases with long-term follow-up, systematically delineating the clinical, imaging, and prognostic characteristics across different age groups. The main findings were as follows: (1) The clinical presentation of pediatric HSE exhibited significant age-related heterogeneity. Unilateral facial and/or perioral twitching was an early characteristic sign of HSE in children under 5 years of age and was closely associated with extensive fronto-insular involvement and long-term language impairment. (2) A low acute GCS score and a longer total duration of seizure activity were independently associated with poor long-term neurological outcomes. (3) The incidence of PE was as high as 53.6%, more than half of which were drug-refractory; 17.9% of patients developed subsequent anti-NMDAR encephalitis, with its incidence increasing significantly with age, representing a core challenge in the long-term management of survivors. To our knowledge, this longitudinal cohort study provides a detailed characterization of age-stratified clinical and imaging features in 56 pediatric HSE patients with long-term follow-up, contributing to the growing body of literature on this condition (8, 11, 14). The age-stratified observations in this study should be interpreted as exploratory and descriptive rather than confirmatory, given the limited sample sizes in the older age subgroups. Nevertheless, these findings provide clinically valuable insights into the distinct presentation patterns of HSE across the pediatric age spectrum, a topic that has been insufficiently characterized in the literature.

(1) Clinical characteristics of HSE in children exhibit age-related heterogeneity, and facial and/or perioral twitching should be recognized as an early diagnostic feature.

In our cohort, the median age at onset was 2.09 years, and 60.7% of patients were children under 3 years of age, which is consistent with previous studies (6, 7, 11, 15), confirming that young children are a highly susceptible population for HSE. Unlike the typical manifestations in adults (2, 16, 17), the predominant presenting symptoms in our pediatric patients were fever, seizures, and impaired consciousness. Symptoms that rely on subjective expression and mature cognitive function, such as headache and memory impairment, became more common after school age. We acknowledge that neuropsychiatric symptoms, including headache and language-related complaints, are particularly challenging to assess in young children due to altered consciousness and limited cognitive capacity to articulate subjective experiences. Therefore, these findings may be underestimated in the infant/toddler age group, and our conclusions regarding age-related differences in these symptoms should be interpreted with this limitation in mind. These differences suggest that clinical evaluation of pediatric HSE should pay close attention to non-specific manifestations. Particularly in young children who are unable to fully articulate their symptoms, seizures may be the presenting feature (17).

Our study identified a highly noteworthy clinical feature: unilateral facial and/or perioral twitching occurred in 33.9% of patients during the acute phase, and all such cases were in children under 5 years of age. The incidence was 35.3% in the infant/toddler group and as high as 70.0% in the preschool group. This manifestation corresponds to early features of opercular syndrome (OS) (17–20). OS is a form of cortical pseudobulbar palsy characterized by alterations of the facio-glosso-pharyngo-laryngeal musculature, leading to loss of voluntary control of these muscles. Studies suggest that OS may be one of the early neurological manifestations of pediatric HSE. From a neuroanatomical perspective, our study found that the proportions of frontal lobe and left insular lobe involvement were higher in the facial/perioral twitching group than in the non-twitching group, supporting the localizing value of the opercular cortex as the origin of motor seizures. This sign has received less attention in previous studies of adult HSE, possibly because adult HSE often presents initially with temporal lobe symptoms such as memory impairment and abnormal mental/behavioral status. The twitching group had a higher rate of severe long-term language impairment at follow-up, consistent with previous reports that pediatric HSE patients with acute-phase OS often have persistent, severe dysarthria and dysphagia during the convalescent phase, directly related to frontal opercular involvement (20). Although there was no statistically significant difference in poor overall prognosis between the two groups, the high incidence of language impairment has a profound impact on the quality of life of preschool-aged children, suggesting that early language rehabilitation intervention should be implemented for this population. Clinicians should recognize that in infants and young children, unexplained facial/perioral twitching, especially when accompanied by fever or other signs of infection, warrants urgent evaluation for HSE.

We acknowledge that the preschool group, which had the highest incidence of facial/perioral twitching, also exhibited the greatest overall severity (lowest GCS, highest lesion burden, and universal bilateral involvement). Therefore, the observed association between twitching and fronto-insular involvement/language impairment may be confounded by overall disease severity. Notably, twitching was not significantly associated with the composite poor prognosis (mRS > 2, *p* = 0.183; Table 1), suggesting that this sign should be interpreted as a localizing clinical feature rather than an independent prognostic predictor. Future studies with larger sample sizes and adjustment for severity are needed to clarify its independent value.

Our MRI findings are broadly consistent with those reported by Pham et al. in a cohort of 37 pediatric HSE patients (21). In both studies, temporal lobe, insular, and parietal lobe involvement were most frequent, confirming the limbic predilection of HSE even in young children. However, we observed higher thalamic involvement (85.7% vs. 61%) and lower hemorrhage (23.2% vs. 42%), which may reflect differences in MRI sequences or disease severity. Leonard et al. demonstrated that extratemporal involvement is more common in young children, attributed to hematogenous spread via an immature blood brain barrier (22). Our data strongly support this. Previous case reports described OS as a rare presentation of pediatric HSE (18). White matter hyperintensities (51.8%) were also common, contrasting with the predominantly gray matter involvement described in adult HSE and supporting distinct pathophysiological mechanisms in children. Pham et al. identified occipital lobe involvement as a predictor of poor outcome at discharge. In contrast, our long-term study found that lesion burden (≥7 lobes) and bilateral involvement were more strongly associated with poor prognosis, suggesting that the cumulative extent of injury may better predict long-term outcome than any single lobe. These comparisons highlight the need for standardized imaging protocols in future multicenter studies.

(2) Severe impairment of consciousness during the acute phase and prolonged total duration of seizure activity are independently associated with poor long-term prognosis.

In this study, 73.2% of patients had a poor prognosis, with intellectual disability (76.8%), language impairment (64.3%), and PE (53.6%) being the most common sequelae, consistent with previous reports (11, 23). Multivariate logistic regression analysis showed that a low acute GCS score and a longer total duration of seizure activity were independently associated with poor prognosis in this exploratory analysis. The GCS score, as an objective indicator of the severity of impaired consciousness, is directly associated with dysfunction of the brainstem reticular activating system and widespread bilateral hemispheric lesions. A low GCS score has been correlated with poor outcomes in patients with encephalitis (2, 24–27) and is a risk factor for the development of anti-NMDAR encephalitis secondary to HSE. Clinicians should therefore provide timely intervention and regular follow-up for pediatric HSE patients with low GCS scores in order to facilitate early identification of secondary anti-NMDAR encephalitis and improve prognosis. In this study, the total duration of seizure activity was measured in days, reflecting recurrent or persistent seizures rather than the duration of a single seizure episode. Such recurrent seizures may cause cumulative brain damage through excitotoxicity, metabolic exhaustion, and sustained neuroinflammatory cascades (28, 29), and their detrimental effect may exceed that of a single episode of SE. In clinical practice, seizure control in pediatric HSE should not be limited to terminating SE; the goal should be a seizure-free state during the acute phase, with close EEG monitoring and active adjustment of ASMs.

(3) PE and anti-NMDAR encephalitis represent core challenges in the long-term management of HSE survivors.

The literature reports that 40–60% of patients with HSE develop PE (14, 28). In our cohort, the incidence of PE was 53.6%, similar to previous reports. However, 63.3% of these cases were drug-refractory, 14 patients (25%) presented with epileptic spasms, and two progressed to LGS, indicating that post-HSE epilepsy is highly refractory. Anti-NMDAR encephalitis developed in 17.9% of patients, with the incidence increasing significantly with age and a median interval of 31.5 days, consistent with previous reports (1, 24), shorter than that in adults (30). Older age is associated with a more mature immune system and a greater propensity for autoimmune reactions. The proportion of patients developing secondary anti-NMDAR encephalitis in our study was higher than in some previous reports (11, 24), which may be attributed to the longer follow-up duration and more systematic autoantibody screening in our study. During the convalescent phase of HSE, particularly within 4 weeks after disease onset, clinicians should remain vigilant for manifestations such as abnormal mental/behavioral status, movement disorders, or seizure recurrence and promptly perform CSF testing for NMDAR antibodies.

This study has several limitations. First, this was a single-center study with a limited overall sample size, and the subgroup analyses, particularly the preschool and school-age and older groups, were particularly underpowered for definitive comparisons. Therefore, the age-related observations should be interpreted as descriptive and hypothesis-generating rather than confirmatory. External validation in larger multicenter cohorts is warranted. Second, handedness was not recorded. Therefore, a causal relationship between left-sided lesions and language impairment could not be established; it was inferred only on the basis of the dominant hemisphere hypothesis. Third, the threshold of more than seven DWI-involved lobes and a seizure duration longer than 5 days was derived from our own cohort data using ROC/Youden optimization. Although these cut-offs may be useful for descriptive stratification, they have not been externally validated and should be interpreted with caution. Future studies with larger, independent cohorts are needed to confirm the generalizability of these thresholds. Fourth, anti-NMDAR antibody testing was performed on a symptom-triggered basis rather than systematically in all patients. This symptom-triggered strategy may have led to underestimation of the true incidence of secondary autoimmune encephalitis, particularly in patients with subtle or atypical presentations who may not have triggered testing. Although our sensitivity analyses suggested that differential follow-up did not substantially affect the estimate, systematic antibody screening in all HSE survivors would be required to determine the true incidence. Fifth, the pediatric mRS, while widely used, is not specifically designed to capture subtle cognitive or language deficits in very young children. Furthermore, pre-morbid developmental status was not systematically recorded, which may have confounded outcome attribution. However, the mRS remains the most practical and widely adopted global functional outcome measure in pediatric neurocritical care, allowing comparability with other cohorts. Additionally, although the majority of patients underwent in-person neurological assessments during follow-up, a proportion of evaluations were conducted via telephone interviews. This approach may have introduced some degree of informant bias in the assessment of subtle neurological deficits, such as mild cognitive or language impairments, compared with objective face-to-face clinical examinations. However, the use of the structured mRS, which relies on functional status rather than detailed neuropsychological testing, likely mitigated this limitation to some extent.

## 5 Conclusion

In this exploratory cohort, clinical characteristics, imaging findings, and prognosis of pediatric HSE showed notable age-associated trends. However, given the small subgroup sizes, these findings should be considered hypothesis-generating and require validation in larger prospective cohorts. Facial/perioral twitching is an early clinical sign in young children and is closely associated with long-term language impairment. Low acute GCS score and prolonged total seizure duration are independent risk factors for poor prognosis, highlighting the need for improved consciousness monitoring and seizure control. The risk of HSV-induced anti-NMDAR encephalitis increases with age, requiring long-term vigilance. This study contributes additional evidence to support for the early recognition, stratified management, and long-term follow-up of pediatric HSE.

## Data Availability

The original contributions presented in the study are included in the article/Supplementary material, further inquiries can be directed to the corresponding authors.
