# Supplementary material for: Age-stratified clinical characteristics and long-term neurological outcomes of pediatric herpes simplex virus encephalitis: a longitudinal cohort study of 56 children
Source: Front Neurol. 2026 Jul 23;17:1882046. doi: 10.3389/fneur.2026.1882046 (PMC13442448; doi:10.3389/fneur.2026.1882046)
Supplement: Supplementary file 1 [file Supplementary_file_1.doc]

Supplemental Table 1 Age-stratified characteristics of children with HSE

|  | All children  (*n*=56) | Infancy/toddlers  (*n*=34) | Preschool  (*n*=10) | School-age and older  (*n*=12) | *p* | Bonferroni‑adjusted *p* |
| --- | --- | --- | --- | --- | --- | --- |
| Females, n (%) | 29(51.8) | 17(50) | 5(50) | 7(58.3) | 0.931 | - |
| Age at onset, years, median, IQR | 2.09(0.95-4.96) | 1.26(0.69-1.85) | 3.62(3.29-4.94) | 8.55(7.48-10.24) | <0.001 | - |
| Symptoms at onset | | | | | |  |
| Fever, n (%) | 55(98.2) | 34(100) | 9(90) | 12(100) | 0.179 | - |
| Seizures, n (%) | 50(89.3) | 31(91.2) | 10(100) | 9(75) | 0.168 | - |
| Alteration consciousness, n (%) | 31(55.4) | 16(47.1) | 5(50) | 10(83.3) | 0.090 | - |
| GCS score, median (IQR) | 7(5.25,9) | 7(5.75,9) | 6(4.75,7) | 8.5(6.25,9.75) | 0.026 | **-** |
| Headache, n(%) | 4(7.1) | 0(0) | 0(0) | 4(33.3) | 0.002 | **-** |
| Vomiting, n (%) | 10(17.9) | 4(11.8) | 2(20) | 4(33.3) | 0.215 | - |
| Status epilepticus, n (%) | 20(35.7) | 16(47.1) | 1(10) | 3(25) | 0.077 | - |
| facial/perioral twitching, n(%) | 19(33.9) | 12(35.3) | 7(70)[7/10] | 0(0) | 0.001 | **-** |
| Neuropsychiatric symptoms, n (%) | 6(10.7) | 3(8.8) | 0(0) | 3(25) | 0.168 | - |
| Hemiparesis, n (%) | 30(53.6) | 20(58.8) | 9(90)[9/10] | 5(41.7) | 0.055 | - |
| Dysphagia, n(%) | 37(66.1) | 23(67.6) | 7(70)[7/10] | 7(58.3) | 0.853 | - |
| Language disorders, n(%) | 38(67.9) | 19(55.9) | 9(90)[9/10] | 10(83.3) | 0.050 | - |
| Memory impairment, n (%) | 14(25) | 5(14.7) | 2(20) | 7(58.3) | 0.002 | **-** |
| ICU admission, n (%) | 42(75) | 25(73.5) | 9(90)[9/10] | 8(66.7) | 0.429 | - |
| Abnormal MRI (DWI) | | | | | |  |
| Number of lobes involved, median (IQR) | 8(6,10) | 8(5,10) | 10(8.5,11) | 6.5(6,8.75) | 0.019 | **-** |
| Lesions ≥7 lobes, n (%) | 26(46.4) | 16(47.1) | 8(80)[8/10] | 2(16.7) | 0.012 | 0.108 |
| Bilateral, n(%) | 42(75) | 27(79.4) | 10(100)[10/10] | 5(41.7) | 0.004 | 0.036 |
| Hemorrhage component, n (%) | 13(23.2) | 5(14.7) | 4(40)[4/10] | 4(33.3) | 0.171 | 1.000 |
| White matter hyperintensities | 29(51.8) | 19(55.9) | 6(60)[6/10] | 4(33.3) | 0.394 | - |
| Meningeal hyperintensity | 6(10.7) | 4(11.8) | 1(10)[1/10] | 1(8.3) | 1.000 | - |
| Midline shift | 6(10.7) | 3(8.8) | 2(20)[2/10] | 1(8.3) | 0.604 | - |
| Frontal lobe, n(%) | 46(82.1) | 27(79.4) | 10(100)10 | 9(75) | 0.583 | 1.000 |
| Temporal lobe, n(%) | 50(89.3) | 29(85.3) | 9(90)[9/10] | 12(100) | 0.127 | 1.000 |
| Parietal lobe, n (%) | 49(87.5) | 29(85.3) | 9(90)[9/10] | 11(91.7) | 0.373 | 1.000 |
| Occipital lobe, n(%) | 18(32.1) | 9(26.5) | 5(50)[5/10] | 4(33.3) | 0.290 | 1.000 |
| Insula, n(%) | 39(69.6) | 20(58.8) | 8(80)[8/10] | 11(91.7) | 0.016 | 0.144 |
| Thalamus, n(%) | 48(85.7) | 28(82.4) | 10(100)[10/10] | 10(83.3) | 0.451 | 1.000 |
| Outcome | | | | | |  |
| mRS>2, n(%) | 41(73.2) | 26(76.5) | 9(90)[9/10] | 6(50) | 0.124 | - |
| PE, n(%) | 30(53.6) | 19(55.9) | 5(50)[5/10] | 6(50) | 0.934 | - |
| Epileptic spasms, n (%) | 14(25) | 9(26.5) | 3(30)[3/10] | 2(16.7) | 0.828 | - |
| Intellectual disability, n (%) | 43(76.8) | 27(79.4) | 8(80)[8/10] | 8(66.7) | 0.671 | - |
| Language disorders, n(%) | 36(64.3) | 21(61.8) | 9(90)[9/10] | 6(50) | 0.115 | - |
| Motor disability, n (%) | 23(41.1) | 15(44.1) | 6(60)[6/10] | 2(16.7) | 0.112 | - |
| Anti-NMDAR encephalitis, n (%) | 10(17.9) | 2(5.9) | 2(20)[2/10] | 6(50) | 0.002 | **-** |

Data are presented as n (%) or median (IQR). Lobe involvement indicates involvement of either side (left or right). Detailed left/right breakdown is available from the corresponding author upon reasonable request. Percentages in square brackets indicate the actual numerator/denominator for subgroups with small sample sizes to facilitate accurate interpretation. *p* were calculated using chi-square test, Fisher's exact test, Kruskal-Wallis test, or Mann-Whitney U test as appropriate.

Bonferroni correction was applied to the 9 MRI related variables (frontal, temporal, parietal, occipital, insular, thalamus, lesions ≥7 lobes, bilateral lesions, and hemorrhage component), with corrected significance threshold α = 0.05/9 = 0.0056. Adjusted *p* values were calculated as *p* × 9; values >1 are reported as 1.000. No lobe-specific variables reached statistical significance at the corrected threshold.

All subgroup and lobe-specific comparisons are exploratory; findings with Bonferroni adjusted *p* < 0.0056 were considered significant at the corrected level. Variables without adjusted *p* values are presented as descriptive exploratory analyses without multiple comparison correction. All such findings require validation in independent cohorts.

The figure shows FLAIR sequence template images: from left to right, sagittal, coronal, and transverse sections, overlaid with color-coded maps indicating the anatomical lobar divisions of the brain. A: Anterior. S: Superior. P: Posterior. L: Left. R: Right.

Supplemental Figure 1 Anatomical atlas used for radiological analysis.
